# Benzimidazole-Quinoline Hybrids: Synthesis and Antimicrobial Properties

**DOI:** 10.3390/ph19010180

**Published:** 2026-01-20

**Authors:** Maria Marinescu

**Affiliations:** Department of Inorganic Chemistry, Organic Chemistry, Biochemistry and Catalysis, Faculty of Chemistry, University of Bucharest, Soseaua Panduri, 030018 Bucharest, Romania; maria.marinescul@chimie.unibuc.ro

**Keywords:** benzimidazole, quinoline, hybrids, antibacterial, antifungal, SAR studies, cytotoxic, anticancer

## Abstract

**Background**: Heterocyclic compounds are particularly important in medicinal chemistry. With a range of therapeutic uses, benzimidazoles and quinolines are both key heterocycles in medicinal chemistry. A number of hybrid heterocyclic compounds have been reported in recent years because they typically have better therapeutic properties than single heterocyclic rings. **Methods**: A literature search was conducted across relevant scientific literature from peer-reviewed sources, using keywords, including “benzimidazole”, “quinoline”, “benzimidazole-quinoline hybrids”, “antibacterial”, “antifungal”, “antimalarial” and “hybrid complexes”. **Results**: This review summarizes the synthetic methodologies for benzimidazole–quinoline hybrids, benzimidazole– quinolinones, and benzimidazole–quinoline metal complexes, along with their antimicrobial and antimalarial activities and the reported structure–activity relationship (SAR) studies. The importance of halogen substitution, particularly with chlorine and fluorine atoms, as well as the structure of the linker between the benzimidazole and quinoline rings—specifically chain length, the presence of oxygen, sulfur, or nitrogen atoms, and heterocyclic moieties—is highlighted. A series of benzimidazole–quinoline hybrids exhibit antimalarial and antitrypanosomal activities or show enhanced antimicrobial properties due to the incorporation of a five-membered heterocycle in addition to the two existing heterocyclic rings. Notably, several hybrids from different compound series exhibit very low minimum inhibitory concentrations (MICs) in the range of 1–8 µg/mL, along with low cytotoxicity, supporting their potential for further investigation as antimicrobial agents. **Conclusions**: This review summarizes the synthetic methods, medicinal properties, and structure–activity relationship (SAR) studies of benzimidazole–quinoline hybrids reported between 2002 and 2026.

## 1. Introduction

**Figure 1:**
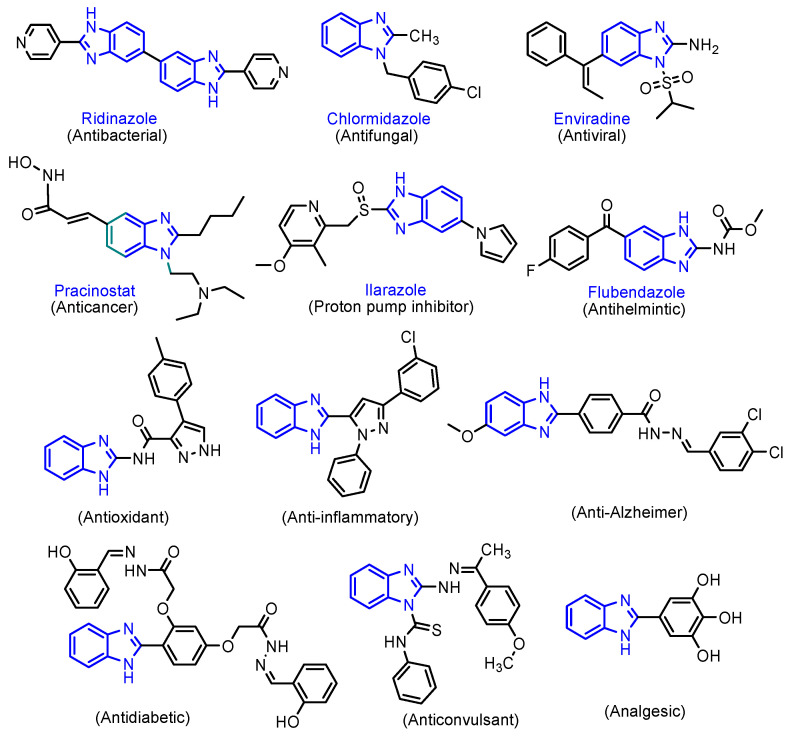
Benzimidazole derivatives exhibiting various medicinal properties.

Heterocyclic compounds are central to medicinal chemistry [1,2], and extensive studies have resulted in the remarkable advancement of heterocycles with pharmaceutical potential [3,4]. Additionally, heterocyclic hybrids have been shown to possess enhanced medicinal properties relative to single heterocyclic systems [5,6], prompting increased efforts toward the synthesis of molecules with multiple heterocyclic rings [7,8,9]. In particular, the benzimidazole ring exhibits a wide range of therapeutic properties [10,11,12,13], including antibacterial [14], antifungal [15], antiviral [16], anticancer [17], antiulcer [18], antihelmintic [11], antioxidant [19], anti-inflammatory [20], analgesic [21], antidiabetic [22], anticonvulsant [23], anti-Alzheimer [24], and neuroprotective activities [25] (Figure 1). Table 1 lists the FDA-approved benzimidazole-based drugs. In addition, several recent reviews have highlighted the notable antimicrobial properties of benzimidazole-based hybrids, including benzimidazole–coumarins [26], benzimidazole–pyrazoles [27], benzimidazole–triazoles [28], benzimidazole–quinazolines [29], and benzimidazole–pyrimidines [30].

**Figure 2:**
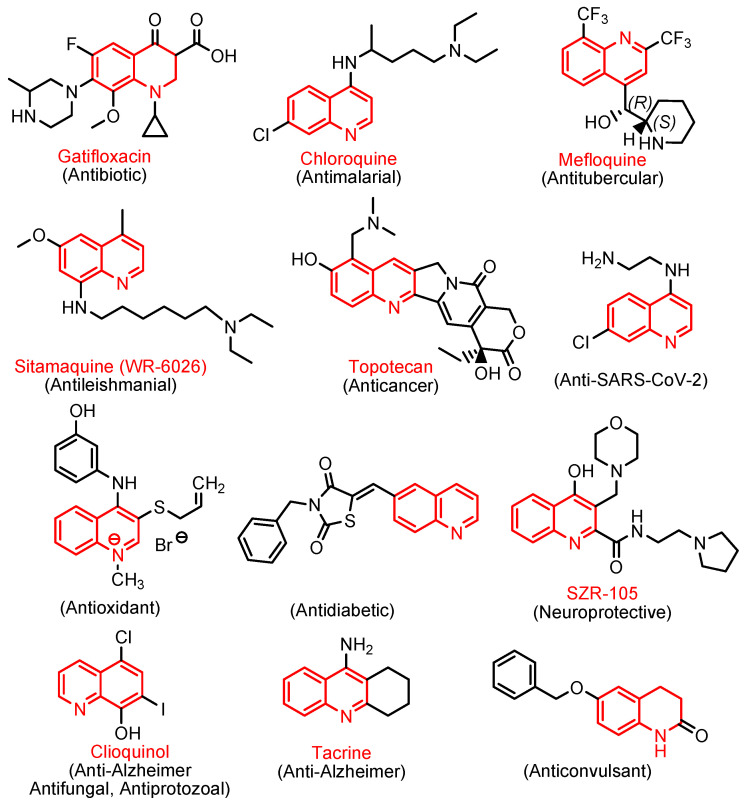
Quinoline derivatives exhibiting various medicinal properties.

Quinoline is a versatile heterocycle in medicinal chemistry, valued for its broad spectrum of therapeutic activities, including antimicrobial [31], antimalarial [32], antitubercular [33], antileishmanial [34], anticancer [35], antioxidant [36], anti-SARS-CoV-2 [37], antidiabetic [38], antidepressant [39], and anticonvulsant properties [40] (Figure 2). Table 2 lists the FDA-approved quinoline-based drugs. Moreover, recent studies have reported quinoline-based hybrids with remarkable medicinal potential, such as quinoline–pyrazole [41], quinoline–triazole [42], quinoline–oxadiazole [43], and quinoline–piperazine derivatives [44].

In recent years, a series of benzimidazole-quinoline hybrids have been synthesized as therapeutic candidates, with unique antibacterial and antifungal pharmaceutical properties, which could make them new therapeutic agents for the treatment of infections with various pathogens. While most studies focus on their antibacterial properties, the literature also reports anticancer [45,46,47,48], antidiabetic [49], anticonvulsant [50], anticoagulant [49], and anti-inflammatory activities [51,52]. Furthermore, benzimidazole–quinoline hybrids have demonstrated potential utility as biomarkers in cancer diagnosis [53].

Among the synthetic strategies for benzimidazoles described in recent literature are the Phillips–Ladenburg reaction, involving the condensation of 1,2-diaminobenzenes with carboxylic acids; the Weidenhagen reaction, which couples 1,2-diaminobenzenes with aldehydes or ketones; and the rearrangement of quinoxalinones [27,54].

Among the reported synthetic approaches for quinolines are the Friedlander synthesis, involving the condensation of 2-aminobenzaldehydes with ketones; the Conrad–Limpach synthesis, based on the condensation of aniline derivatives with β-ketoesters; the Doebner–Miller reaction, in which aldehydes or α,β-unsaturated ketones react with aniline; and the intramolecular cyclization of acetanilides [55].

Accordingly, the objective of this review is to provide an overview of the synthetic strategies, antimicrobial properties, and SAR analyses of benzimidazole–quinoline hybrids reported in the literature.

A comprehensive literature search was conducted across multiple scientific databases, including PubMed, Scopus, and ScienceDirect, as well as publishers’ platforms such as ACS Publications, MDPI, Springer, the Royal Society of Chemistry, and Taylor & Francis. Search terms included “quinoline,” “benzimidazole,” “benzimidazole–quinoline hybrids,” “Conrad–Limpach reaction,” “antibacterial,” “antifungal,” “SAR studies,” and other relevant therapeutic properties. Priority was given to publications from the last ten years.

## 2. Benzimidazole-Quinoline Hybrids with Antimicrobial Properties

**Scheme 1:**
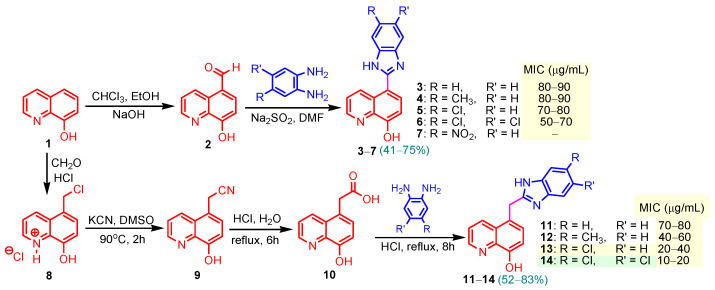
Synthesis of antibacterial hybrids **3**–**7** and **11**–**14**.

El Faydy et al. (2022) [56] reported the synthesis of two series of benzimidazole–quinoline hybrids, **3**–**7** and **11**–**14**, starting from quinolin-8-ol **1** (Scheme 1). Reimer–Tiemann formylation of **1** with chloroform and sodium hydroxide in ethanol afforded 8-hydroxyquinoline-5-carbaldehyde **2**, which subsequently underwent a Weidenhagen reaction with disubstituted benzene-1,2-diamines to yield hybrids **3**–**7**. The second reaction sequence involved chloromethylation at the “5” position of **1**, followed by conversion to the corresponding nitrile **9** by nucleophilic substitution with potassium cyanide in dimethylsulfoxide (DMSO) at 90 °C. Acidic hydrolysis of nitrile **9** under reflux for 6 h yielded 2-(8-hydroxyquinolin-5-yl)acetic acid **10**, which subsequently underwent the Phillips-Ladenburg reaction to afford hybrids **11**–**14**.

**Figure 3:**
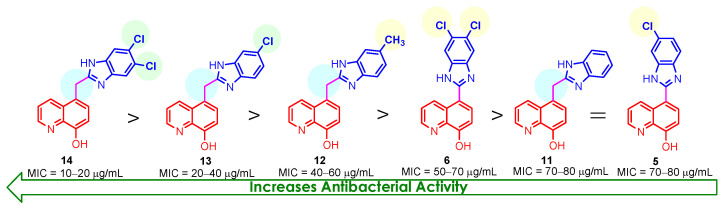
Antibacterial activity variation in compounds **5**, **6**, **11**–**14** based on substituent presence.

The antibacterial activity of the compounds was evaluated by determining the minimum inhibitory concentrations (MICs) of the hybrids against four bacterial strains: *B. subtilis*, *S. aureus*, *E. coli*, and *E. ludwigii*. Analysis of the MIC values revealed that the second series of compounds exhibited enhanced antibacterial activity. The presence of substituents at the “5”and “6” positions of the benzimidazole ring plays a crucial role in enhancing biological activity through their electronic effects. In particular, the –Chloro substituent on benzimidazole ring contributes via its electron-releasing electromeric effect, with the number of chlorine atoms exerting a greater influence than the inductive effect of the CH_3_ group. Consequently, antimicrobial activity decreases in the following order: **14** (two chlorine atoms) > **13** (two chlorine atoms) > **12** (one CH_3_ group). Accordingly, compounds **3** and **11**, which are unsubstituted on both the benzimidazole and quinoline rings, exhibited only modest activity, with MIC values ranging from 70 to 90 µg/mL, as illustrated in Scheme 1. Compound **12**, which contains a methylene group at the position “5” of the quinoline ring in addition to its analogue, hybrid **4**, exhibited notably improved activity, with MIC values of 40–60 µg/mL. The most pronounced enhancement in antibacterial activity was observed for compound **13**, featuring a chlorine atom at the position “5” of the benzimidazole ring, with MIC values of 20–40 µg/mL. Hybrid **14**, containing two chlorine atoms, displayed antibacterial activity comparable to that of the standard Nitroxoline, with MIC values of 10–20 µg/mL The presence of the methylene linker between position “5” of quinoline and position “2” of benzimidazole plays a particularly important role, even surpassing the effect of chlorine substitution on the benzimidazole ring, according to structure–activity relationship (SAR) analysis. This is reflected in the variations in antibacterial activity shown in Figure 3, which is correlated with increased molecular flexibility and greater receptor contact [56]. The role of chlorine in enhancing antibacterial activity is consistent with findings previously reported by other researchers [57,58].

**Scheme 2:**
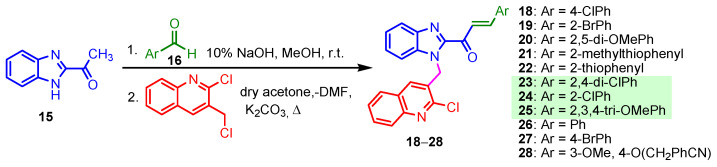
Synthesis of antibacterial chalcone hybrids **18**–**28**.

**Figure 4:**
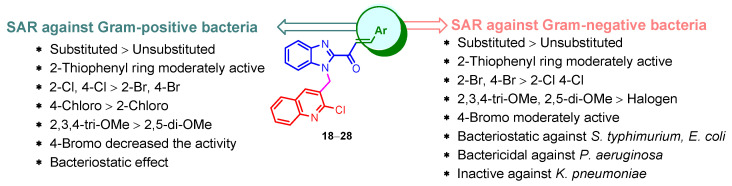
Structure–activity relationship (SAR) of the chalcone hybrids **18**–**28**.

Karmur et al. reported the synthesis of hybrids **18**–**28** via a two-step procedure starting from benzimidazole-ethanone **15**: (i) base-catalyzed condensation with aromatic aldehydes at room temperature to form intermediate chalcones, followed by (ii) reaction with 2-chloro-3-(chloromethyl)quinoline **17** upon heating in anhydrous acetone and dimethylformamide (DMF) (Scheme 2). The antibacterial activity of hybrids **18**–**28** was evaluated against Gram-positive strains (*Staphylococcus aureus*, *Bacillus cereus*, and *Corynebacterium rubrum*) and Gram-negative bacteria (*Salmonella typhimurium*, *Escherichia coli*, *Klebsiella pneumoniae*, and *Pseudomonas aeruginosa*), using tetracycline as the reference standard. Compounds **23**, **24**, and **25** were the most active members of the series against *P. aeruginosa* and *E. coli*, exhibiting an average zone of inhibition (ZoI) of 28 mm, compared with 15.5 mm for tetracycline under the same conditions. With the exception of compounds **19** and **20**, which showed moderate inhibition against *S. typhimurium* with ZoI values of 21.5 and 23.5 mm, respectively, the remaining hybrids were largely inactive against other Gram-negative bacteria, such as *K. pneumoniae* and *S. typhimurium*. The structure–activity relationship (SAR) analysis of hybrids **18**–**28** is summarized in Figure 4. 

The results are consistent with those reported in other studies. Specifically, (1) enhanced antimicrobial activity is observed in the presence of chlorine atoms at the “2” and “4” positions in a phenyl ring (**23**, **24**). (2) Bromine substitution on phenyl ring exhibits opposite effects on Gram-positive and Gram-negative bacteria: it reduces biological activity against Gram-positive strains due to steric hindrance, while enhancing activity against Gram-negative bacteria (**27**), surpassing even that observed for chlorine-substituted derivatives (**18**, **24**). This behavior may be correlated with variations in the lipophilicity of bromine-substituted molecules, which favor activity against Gram-negative bacteria. (3) Substitution with three methoxy groups (**25**) results in improved antimicrobial activity against both bacterial types, followed by substitution with two methoxy groups (**20**); both are more effective than chlorine substitution. This enhancement can be attributed to the cumulative electron-releasing electromeric effect of the three methoxy groups, as well as to the stereochemical features of the compounds that enable specific interactions at the receptor protein binding site. Molecular docking studies further indicated that compounds **23**, **24**, and **25** effectively inhibit TyrRS or DNA gyrase, correlating with their potent antibacterial activity [59].

**Scheme 3:**
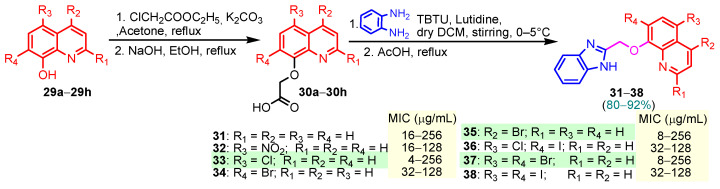
Synthesis of antibacterial hybrids **31**–**38**.

**Figure 5:**
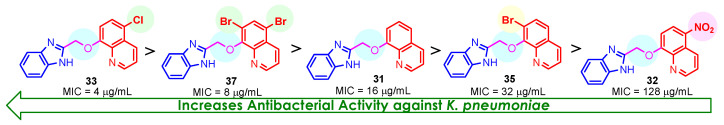
Variation in antibacterial activity of hybrids **31**–**33**, **35**, and **37** as a function of substituent presence.

Prashanth et al. (2025) reported the synthesis of benzimidazole–quinoline hybrids **31**–**38** via a four-step protocol starting from 2,4,5,7-tetrasubstituted quinolin-8-ols (**29a**–**29h**), as depicted in Scheme 3 [60,61]. The antibacterial activity of these compounds was evaluated against Gram-positive bacteria, including methicillin-resistant *Staphylococcus aureus* (MRSA), *Staphylococcus simulans*, *Staphylococcus epidermidis*, *Staphylococcus aureus*, and *Enterococcus faecalis*, as well as Gram-negative bacteria, namely *Klebsiella pneumoniae*, *Escherichia coli*, *Pseudomonas aeruginosa*, *Proteus mirabilis*, and *Salmonella typhi*, using Ciprofloxacin and Vancomycin as reference standards. Disc diffusion assays showed that all eight hybrids were active, exhibiting inhibition zones of 8–31 mm against both Gram-positive and Gram-negative bacteria, comparable to those of the reference antibiotics Cancomycin and Ciprofloxacin. Notably, compound **37**, bearing two bromine atoms on the quinoline ring, displayed outstanding antibacterial activity, with the lowest minimum inhibitory concentration (MIC) of 8 µg/mL against *Klebsiella pneumoniae*. Compound **34**, bearing a single bromine substituent, exhibited lower antibacterial activity than hybrid **37**, with MIC values ranging from 32 to 128 µg/mL. This result is consistent with the findings of a previous study, which demonstrated that the presence of bromine atoms significantly enhances antibacterial activity against Gram-negative bacteria. However, it also complements the previous work, as the bromine atoms in that study were positioned on a phenyl ring of a chalcone, whereas in the current study, bromine is located on the phenyl ring of quinoline. In contrast, compound **33**, containing a chlorine atom, showed a distinct antibacterial profile, displaying the lowest MIC of 4 µg/mL against *K. pneumoniae* and an MIC of 8 µg/mL against *E. coli*. In this case, the result for molecule **33** is both consistent with and complementary to the previous study. It confirms that the best antimicrobial activity is observed when a chlorine atom is present, regardless of its position—whether on the quinoline phenyl ring or on a separate phenyl group, as seen in the chalcone structure. As illustrated in Figure 5, antibacterial activity against *K. pneumoniae* increases depending on the nature and position of the substituent on the quinoline ring. Specifically, the presence of 5-chloro (**33**) and 5,7-dibromo (**37**) substituents enhances biological activity compared to the unsubstituted hybrid **31**, whereas 7-bromo (**35**) and 5-nitro (**32**) substituents reduce activity relative to compound **31**. The results of docking studies were found to be consistent with the experimental results and demonstrated the significance of the quinoline ring system and nitrogen atoms in the biological activity of the compounds as potential antibacterial drugs [60].

**Scheme 4:**
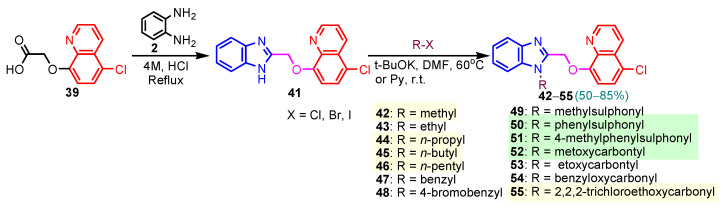
Synthesis of antimicrobial hybrids **42**–**55**.

**Scheme 5:**
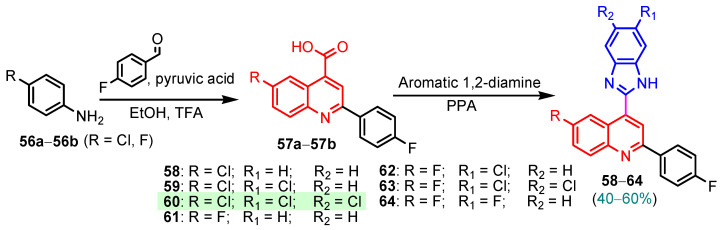
Synthesis of antimicrobial hybrids **58**–**64**.

**Scheme 6:**
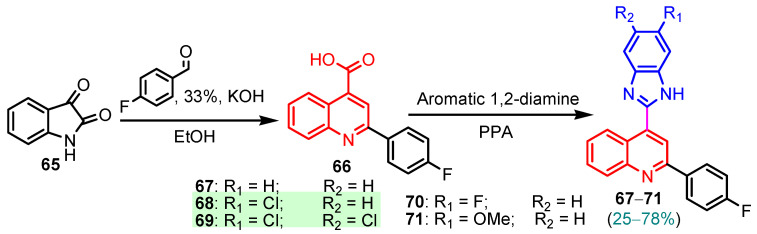
Synthesis of antimicrobial hybrids **67**–**71**.

**Figure 6:**
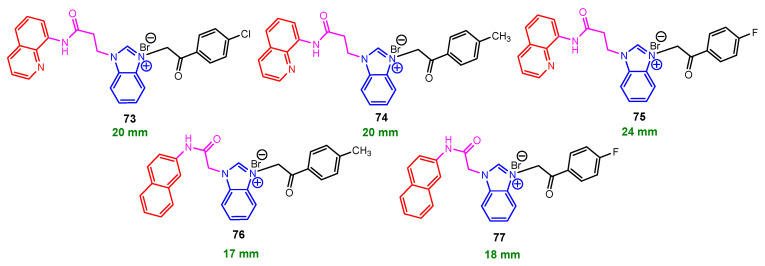
Diameter of inhibition zone of compounds **73**–**77** against *E. coli*.

For compounds **32**, **34**, **35**, and **37**, which showed the lowest binding energies and formed multiple hydrogen bonds with important amino acid residues in the target protein’s active site, especially ASP81 and ARG144, in silico docking simulations revealed favorable binding affinity scores and well-defined hydrogen bond interactions [61]. Hybrids with similar structures exhibit comparable potency ranges for optimal activity. Accordingly, when comparing the two series of compounds, **3**–**14** and **31**–**38**, the best MIC values fall within the ranges of 10–20 µg/mL and 8–16 µg/mL, respectively. Chaudhari and Rindhe reported the synthesis of benzimidazole–quinoline hybrids **42**–**55** via a two-step protocol starting from 2-(5-chloroquinolin-8-yloxy)acetic acid **39** and benzene-1,2-diamine **40**, proceeding through intermediate **41**. The reactions were carried out in dimethylformamide (DMF) at 60 °C to afford hybrids **42**–**48**, while pyridine (Py) at room temperature was employed for the synthesis of hybrids **49**–**55** (Scheme 4). Compounds **41**, **44**, **45**, **46**, **50**, **52**, and **55** exhibited excellent antibacterial activity against *Salmonella typhimurium* and *Staphylococcus aureus*, showing efficacy comparable to that of the reference drugs ciprofloxacin and chloramphenicol. Compared with the reference drug griseofulvin, hybrids **42**, **50**, **51**, and **52** exhibited pronounced antifungal activity against *Aspergillus niger*, while displaying low activity against *Candida albicans*. The results reported by these authors are based solely on in vitro antimicrobial activity determinations, where the zone of inhibition (measured in mm) was assessed at compound concentrations ranging from 50 to 500 µg/mL, without the use of docking or in silico studies [62]. Garudachari et al. (2012) [63] reported the synthesis of two series of benzimidazole–quinoline hybrids, namely **58**–**64** (Scheme 5) and **67**–**71** (Scheme 6), starting from 4-substituted anilines **56a** and **56b** and indoline-2,3-dione **65** via a two-step synthetic route. The first step involved a Pfitzinger reaction leading to the formation of the quinoline ring, followed by a Phillips–Ladenburg reaction to construct the benzimidazole moiety. Hybrids **58**–**64** and **67**–**71** were evaluated for their in vitro antibacterial activity against *Staphylococcus aureus*, *Escherichia coli*, *Xanthomonas* sp., and *Salmonella* sp. (recultured), using Ciprofloxacin as the reference drug, as well as for their in vitro antifungal activity against *Aspergillus niger*, *Aspergillus flavus*, *Aspergillus terreus*, and *Penicillium* sp. (recultured), with Fluconazole as the standard, employing the well plate method (Table 3). Among the screened samples, **58**, **60** and **69** showed excellent inhibition of bacterial growth. Compound **68** exhibited significant antifungal inhibition compared with the reference drug Fluconazole. Compounds **60** and **69** showed enhanced activity, which was attributed to the presence of two chlorine substituents on the benzimidazole ring and a 4-fluorophenyl group at the C “2” position of the quinoline moiety. In the case of compound **68**, the enhanced antifungal activity was attributed to the electronic effects of a 4-fluorophenyl substituent at the C “2” position of the quinoline ring and the electron-releasing effect of the chlorine atom on the benzimidazole scaffold, in full agreement with previously reported research. Additionally, the presence of a fluorine atom in the quinoline skeleton further contributed to the improvement of antimicrobial activity [63]. Overall, the biological evaluation of hybrids **58**–**64** and **67**–**71** confirmed that the incorporation of a substituted benzimidazole pharmacophore at the C “4” position of the quinoline core enhances antimicrobial activity [64]. Diaconu et al. reported the synthesis of compounds **73**–**77** via the reaction of quinoline-8-amine with 3-chloropropanoyl chloride (for **73**–**75**) or 2-chloroacetyl chloride (for **76**–**77**) in chloroform, in the presence of pyridine, at room temperature for 4 h. The resulting intermediates were then reacted with benzimidazole in acetonitrile using sodium hydride for 12 h, followed by the reaction of the benzimidazole–quinoline intermediates with substituted 2-bromo-1-phenylethanone in acetone. All hybrids (**73**–**77**) exhibited superior antibacterial activity against *E. coli* compared with the reference drug Gentamicin, with inhibition zone diameters ranging from 17 to 24 mm, while Gentamicin showed 12 mm (Figure 6) [65]. The antibacterial activity of compounds **73**–**77** was enhanced by electronic effects stemming from the presence of halogen substituents, specifically chlorine and fluorine, as well as methyl groups on the phenyl ring, in agreement with previously reported structure–activity relationships. Better pharmacological characteristics, particularly water solubility and membrane permeability, as well as a more appropriate interaction with the receptor, are caused by the alkyl-amide group that is present between the “8” position of the quinolinic ring and the “1” position of the benzimidazole [66]. El-Gohary and Shaaban (2017) [67] evaluated the antimicrobial activity of hybrids **78** and **79** against *Escherichia coli*, *Bacillus cereus*, *Staphylococcus aureus*, *Candida albicans*, and *Aspergillus fumigatus* 293 (Table 4). Both compounds exhibited enhanced antibacterial activity against *B. cereus* compared with the reference drug Ampicillin (MIC = 1250 µg/mL), showing MICs of 156.25 µg/mL and 312.5 µg/mL, respectively. They also displayed antifungal activity comparable to or exceeding that of Fluconazole (MIC = 2500 µg/mL) against *C. albicans*, with MICs of 1250 µg/mL and 2500 µg/mL, respectively. Additionally, compounds **78** and **79** demonstrated notable antiquorum-sensing activity against *Chromobacterium violaceum* ATCC 12472. When compared to the 5-nitrobenzimidazole analogue **79**, the introduction of the 6-nitroquinolin-5-yl group into the unsubstituted benzimidazole core (compound **78**) resulted in increased activity against all tested strains, as indicated by structure–activity relationship analysis. These findings are consistent with earlier work [56], which demonstrated that the addition of a nitro substituent to the benzimidazole core led to the complete loss of antimicrobial activity [67].

**Scheme 7:**
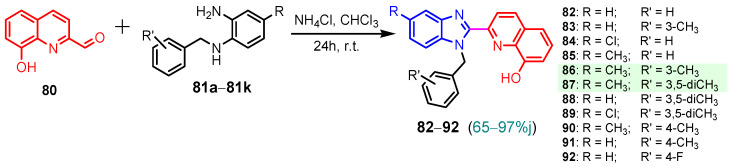
Synthesis of antimicrobial hybrids **82**–**92**.

**Figure 7:**
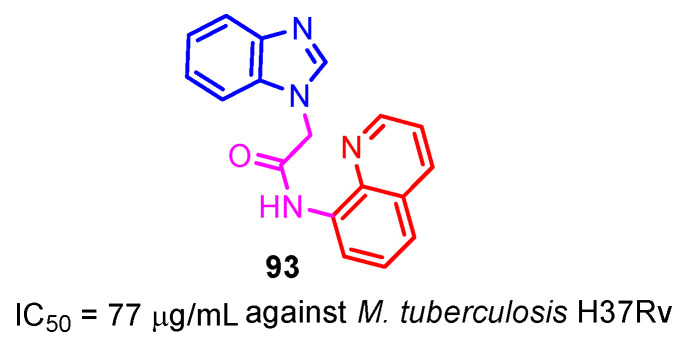
Antimycobacterial hybrid **93**.

Sanwer et al. (2025) reported the synthesis of benzimidazole–quinoline hybrids **82**–**92** via the reaction of 8-hydroxyquinoline-2-carbaldehyde **80** with disubstituted *N*′-benzyl benzene-1,2-diamines **81a**–**81j** in chloroform and ammonium chloride at room temperature for 24 h (Scheme 7) [68]. The hybrids were evaluated for antibacterial activity against *E. coli*, *S. aureus*, *P. aeruginosa*, and *B. subtilis*, and for antifungal activity against *A. niger* and *C. albicans*. The minimum inhibitory concentration (MIC) values for the bacterial strains ranged from 3.9 to 62.5 μg/mL (Table 5). Compound **87** exhibited the highest antibacterial activity, with an MIC of 3.9 μg/mL against both *P. aeruginosa* and *E. coli*. Hybrids **83**, **84**, **86**, **87**, **88**, and **92** showed the strongest activity against *P. aeruginosa* (MIC = 3.9 μg/mL), while compounds **82** and **92** displayed good activity against both *E. coli* and *P. aeruginosa* (MIC = 7.8 μg/mL). Additionally, hybrid **83** showed an MIC of 7.8 μg/mL against *S. aureus*, and **84** displayed the same MIC against *B. subtilis*. The compounds also exhibited notable antifungal activity, with MIC values ranging from 19.2 to 500 μg/mL against *A. niger* and *C. albicans*, with hybrid **86** being the most potent, showing MICs of 22.3 μg/mL and 19.2 μg/mL, respectively. This study makes a significant contribution to structure–activity relationships (SAR) by highlighting the importance of alkyl substituents on the phenyl ring, linked by a methylene bridge to the benzimidazole nitrogen. This effect is even more significant than the electronic influence of the chlorine atom at the 6-position of the benzimidazole ring. The observed effect can be explained by the enhanced lipophilicity of the methyl-substituted compounds, which facilitates better cell membrane penetration and thus improves antimicrobial activity [68]. Mantu et al. reported compound **93**, with an antimycobacterial activity IC_50_ of 77 µg/mL against *Mycobacterium tuberculosis* H37Rv under aerobic conditions (Figure 7). The hybrid derivative **93** has interesting pharmacological characteristics due to its exceptional solubility in microbiological media. The tuberculostatic effect and high solubility of hybrid **93** were associated with the amide structure present in the bridge between the two heterocyclic rings of quinoline and benzimidazole [69].

**Figure 8:**
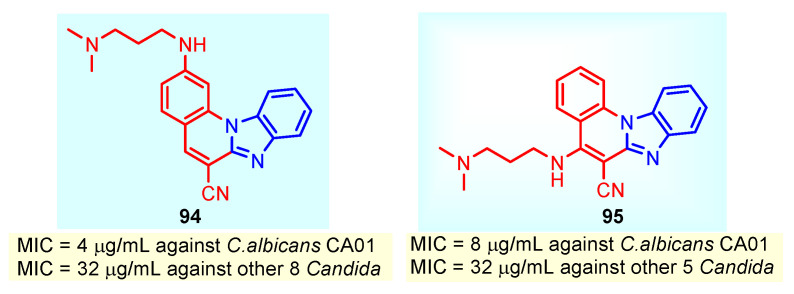
Fluorescent antifungal benzimidazo[1,2-*a*]quinoline hybrids **93** and **94**.

**Scheme 8:**
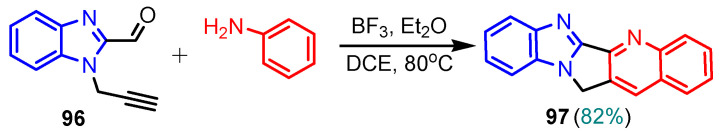
Synthesis of antifungal hybrid **97**.

Perin et al. (2014) reported the synthesis of benzimidazo[1,2-a]quinoline compounds **94** and **95** (Figure 8) through key steps including the aldol condensation of 2-chlorobenzoyl chloride with 2-cyanomethylbenzimidazole in absolute ethanol using piperidine as a base, followed by thermal cyclization of the aldol intermediate in DMF with *t*-KOBu to afford the fused keto benzimidazo–quinoline derivatives [70,71,72,73]. De Souza et al. (2016) demonstrated that these fluorescent benzimidazo[1,2-a]quinolines (**94** and **95**) act as bifunctional agents, capable of detecting and exerting biocidal activity against yeast biofilms on stainless steel surfaces [74]. This approach represents an innovative strategy for preventing contamination in hospital environments. Hybrid **94** exhibited antifungal activity against all nine tested *Candida* strains: three *C. tropicalis* (ATCC 750, ATCC 950, RL17), three *C. albicans* (ATCC 18804, ATCC 24433, CA01), and three *C. parapsilosis* (ATCC 22019, RL33, RL07), with MIC values of 4 mg/mL against *C. albicans* CA01 and 32 mg/mL against the other eight strains. Hybrid **95** was active against six of the strains, with the exceptions of *C. albicans* ATCC 18804, *C. parapsilosis* RL33, and RL07. Fluorescent hybrid **94** functioned as a bifunctional *Candida* biofilm detector and eradicator, successfully visualizing biofilms on stainless steel 304 plates under UV light when sprayed over contaminated surfaces. Therefore, benzimidazole **94** ensures the disinfection of medical and surgical instruments, enhancing the safety of clinical and surgical procedures in hospital environments. The enhanced antimicrobial activity of compounds **94** and **95** can be attributed to the cumulative electronic effects of their substituents: the electron-withdrawing electromeric effect of the nitrile group at the “3” position of the quinoline ring, and the electron-releasing electromeric effect of the amino-alkyl group at the “7” and “4” positions of compounds **94** and **95**, respectively. Additionally, the alkyl group attached to the amino moiety increases the lipophilicity of the compounds, further contributing to their improved activity [74]. Villa et al. synthesized benzimidazole-tethered pyrrolo[3,4-b]quinoline **97** via an intramolecular Povarov reaction between 1-(prop-2-ynyl)-1*H*-benzo[*d*]imidazole-2-carbaldehyde **96** and aniline in 1,2-dichloroethane (DCE), using boron trifluoride diethyl etherate (BF_3_·Et_2_O) as a Lewis acid at 80 °C (Scheme 8). The antifungal activity of compound **97** against 26 clinical fungal pathogens, compared with Fluconazole, is summarized in Table 6. The results indicate that hybrid **97** exhibits superior antifungal activity relative to the standard Fluconazole and effectively inhibits fungal growth without inducing apparent toxicity in mammalian cells. From a structural perspective, the introduction of a methylene bridge between the benzimidazole nitrogen and the carbon at the “3” position of the quinoline, resulting in the formation of an additional pyrrolidine ring in hybrid **97**, offers several advantages: (1) it produces a benzimidazole–quinoline hybrid with an approximately flat surface, enhancing interactions at the receptor site; (2) the pyrrolidine ring provides additional and specific interactions within the receptor protein pocket; and (3) it increases the lipophilicity of the molecule, facilitating cell membrane penetration, which is reflected in superior antimicrobial activity, as confirmed experimentally. These results both support and complement previously reported research [75].

The articles with benzimidazole-quinoline hybrids presented in this subchapter do not refer to in vivo research, that is, only in vitro research is presented, only in some cases, cytotoxicity studies while resistance studies are missing.

The structure–activity relationship (SAR) for benzimidazole-quinoline hybrids with antimicrobial properties can be summarized by the following factors that improve antimicrobial activity:

- The presence of a chlorine atom at the “5” position of the benzimidazole ring [76].
- The presence of two chlorine atoms at the “5” and “6” positions further enhances activity [77].
- Chlorine atoms at the “4” and “2” positions of a phenyl ring.
- The methylene linker between the “5” position of quinoline and the “2” position of benzimidazole [24].
- Bromine substitution on the phenyl ring improves activity against Gram-negative bacteria.
- Substitution with three methoxy groups on a phenyl ring.
- A 4-fluorophenyl group at the C “2” position of the quinoline moiety [78].
- An alkyl-amide group present between the “8” position of the quinoline ring and the “1” position of the benzimidazole ring.
- The absence of a nitro group at the “5” position of the benzimidazole ring.
- The presence of a nitro group at the “6” position of the quinoline ring [79].
- Alkyl substituents on the phenyl ring, linked by a methylene bridge to the benzimidazole nitrogen.
- An alkyl group attached to the amino moiety, which increases the lipophilicity of the compounds.
- Compound **97** from this section exhibited the strongest antimicrobial activity, with MIC values ranging from 0.0625 to 1 µg/mL against 26 clinical fungal pathogens, compared with Fluconazole as the reference drug.

## 3. Benzimidazole-Quinolinolone Hybrids with Antimicrobial Properties

**Figure 9:**
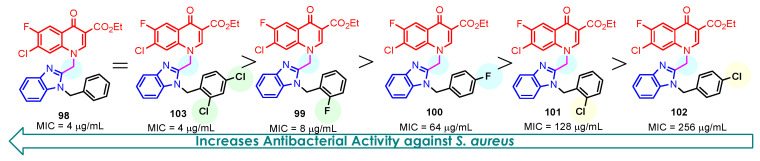
Antibacterial activity variation of hybrids **98**–**103**, based on substituent presence.

**Figure 10:**
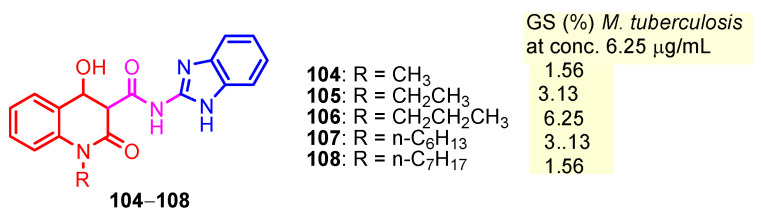
Anti-tuberculosis benzimidazole-quinolones **104**–**108**.

**Figure 11:**
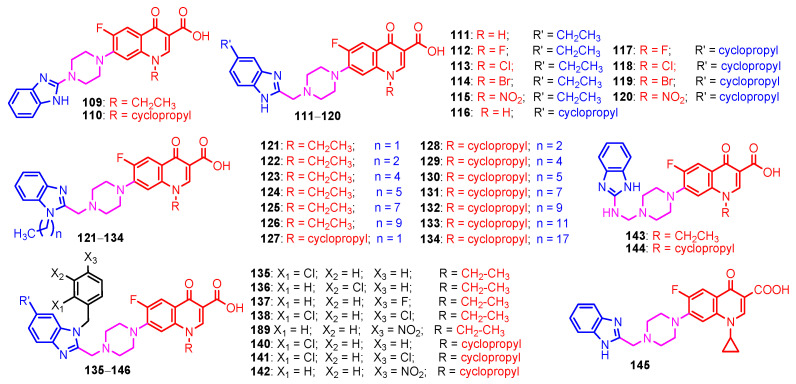
Antibacterial benzimidazole-quinolones **109**–**145**.

**Figure 12:**
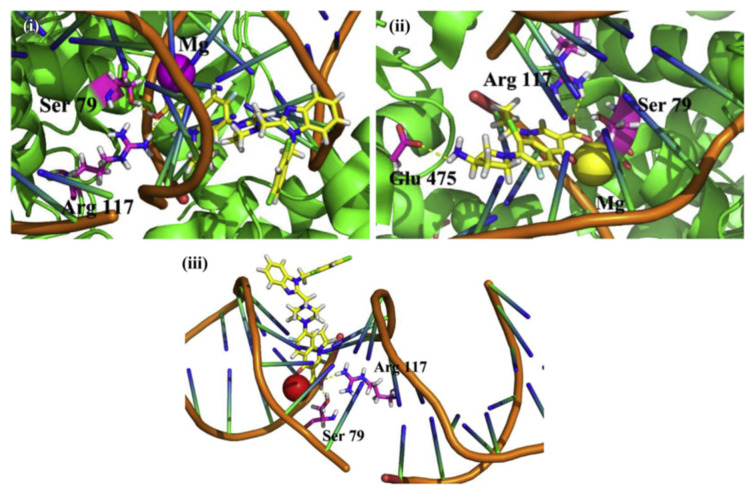
(**i**) Three-dimensional conformation of hybrid **141** docked in topoisomerase IV-DNA complex. (**ii**) Three-dimensional conformation of clinafloxacin docked in topoisomerase IV-DNA complex. (**iii**) Three-dimensional conformation of **141** docked in topoisomerase IV-DNA complex.

Quinolones are recognized for their strong antimicrobial properties, as demonstrated by quinoline-derived antibiotics such as fluoroquinolones, including ciprofloxacin. [80,81]. Accordingly, this subsection highlights literature reports on the antimicrobial activities of benzimidazole–quinoline hybrids [82]. Wang et al. (2018) synthesized benzimidazole- quinolone hybrids **98**–**103** via the reaction of ethyl 7-chloro-6-fluoro-4-oxo-1,4-dihydroquinoline-3-carboxylate with phenyl-substituted 1-benzyl-2-(chloromethyl)-1*H*-benzo[*d*]imidazoles in acetonitrile using potassium carbonate at 70 °C [83]. The in vitro antimicrobial activities for hybrids **98**–**103** were evaluated against five Gram-positive bacteria (MRSA, *Enterococcus faecalis*, *S. aureus*, *S. aureus* ATCC 25923, and *S. aureus* ATCC 29213), six Gram-negative bacteria (*Klebsiella pneumoniae*, *Escherichia coli*, *Pseudomonas aeruginosa*, *Acinetobacter baumanii*, *Pseudomonas aeruginosa* ATCC 27853, *Escherichia coli* ATCC 25922), and five fungal strains (*Candida albicans*, *Candida tropicalis*, *Aspergillus fumigatus*, *C. albicans* ATCC 90023, *Candida parapsilosis* ATCC 22019) using the twofold serial dilution method. Among the compounds, the unsubstituted benzyl derivative **98** exhibited the highest sensitivity toward Gram-positive bacteria, with MIC values ranging from 4 to 64 µg/mL (Table 7). Fluorobenzyl derivatives **99** and **100**, with MIC values of 1–64 µg/mL and 4–128 µg/mL, respectively, exhibited higher antibacterial activity than the chlorobenzyl derivatives **101** and **102**, which showed MICs of 8–256 µg/mL and 32–512 µg/mL, except against *E. coli* and *A. baumannii* strains. The slightly superior activities of **99** and **101** compared with their analogues **100** and **102** suggest that the position of the halogen atoms positively influences bacterial growth inhibition. Notably, the antibacterial activity of compounds **99**–**102** against *S. aureus* gradually decreased depending on the substituents on the phenyl moiety (Figure 9), following the order: unsubstituted phenyl **98** > 2-fluorophenyl **99** > 4-fluorophenyl **100** > 2-chlorophenyl **101** > 4-chlorophenyl **102**. Among the derivatives, dichlorophenyl **103** showed relatively strong inhibition against *S. aureus* (MIC = 4 µg/mL) compared with the monosubstituted analogues Hybrid **99** exhibited a relatively low minimum inhibitory concentration (MIC) of 1 µg/mL, demonstrating 4- and 32-fold greater efficacy against *P. aeruginosa* compared with the reference drugs Clinafloxacin and Norfloxacin. Similar to Norfloxacin, MRSA and *S. aureus* were susceptible to **99**, with an MIC of 8 µg/mL. Moreover, hybrid **99** displayed low cytotoxicity against Hep-2 cells. Molecular docking analysis indicated that **99** could interact with topoisomerase IV–DNA complexes via hydrogen bonds formed with Gly419 and Asp397 residues of topoisomerase IV and the DT15 base of DNA. Preliminary mechanistic studies suggested that hybrid **99** alone did not intercalate into DNA isolated from drug-resistant *P. aeruginosa*, whereas the **99**–Cu^2+^ complex effectively cleaved DNA, potentially inhibiting DNA replication and contributing to its potent bioactivity. Importantly, hybrid **98** was also capable of permeabilizing and disrupting the cell membrane of *P. aeruginosa*. SAR studies reveal that the simultaneous presence of fluorine and chlorine atoms at positions “6” and “7” of the quinoline ring contributes to improved antimicrobial activity. However, the nature of the substituent on the phenyl ring, linked to the methylene bridge of the benzimidazole nitrogen, plays a more significant role. Surprisingly, the unsubstituted phenyl compound **98** emerges as the best antimicrobial agent, along with the 1,4-dichlorosubstituted compound **103**, suggesting that both compounds interact equally well in the protein’s active site and exhibit similar effects. Furthermore, the presence of a fluorine atom at position “2” of the phenyl ring (**98**) is more favorable compared to position “4” (**100**), with a similar trend observed for chloro-substituted analogues, where antimicrobial activity decreases in the order: **101** > **102**. In this case, it appears that the electronic effects resulting from the position of the substituent take precedence over the nature of the substituent, with fluorine substituents demonstrating higher activity than monosubstituted chlorine ones—except for compound **103**, which is energetically favored and has a lipophilicity similar to hybrid **98** [83]. Using the reaction between *N*-substituted ethyl 4-hydroxy-2-oxo-1,2,3,4-tetrahydro quinoline-3-carboxylate and 1*H*-benzo[*d*]imidazol-2-amine, Ukrainets et al. (2011) [84] synthesized anti-tuberculosis benzimidazole–quinoline derivatives **104**–**108** with yields ranging from 80% to 92%. The growth suppression (GS, %) of all compounds was evaluated against *Mycobacterium tuberculosis* H37Rv ATCC 27,294 at a concentration of 6.25 mg/mL. The *n*-propyl hybrid **106** exhibited the highest activity, with a GS of 6.25% (Figure 10). It can be concluded that the presence of a long-chain substituent at the quinolone nitrogen does not promote enhanced tuberculostatic activity, as all tested compounds exhibited weak activity [84]. Zhang et al. (2016) [85] synthesized benzimidazole–quinolone derivatives **109**–**142** from the corresponding quinolone acids or esters and the appropriate benzimidazoles in a basic potassium carbonate medium in acetonitrile at 0–50 °C (Figure 11), with yields ranging from 32% to 88%. Benzimidazole–quinolone hybrids **143** and **144** were prepared via a Mannich reaction starting from 2-aminobenzimidazole, paraformaldehyde, and the corresponding quinolones, with yields of 29.4% and 31.5%, respectively. As shown in Table 8, most hybrids **109**–**143** exhibited good or superior antimicrobial activities compared with the reference drugs Chloromycin, Norfloxacin, Ciprofloxacin, and Clinafloxacin. SAR studies highlight the importance of the methylene-piperazine bridge between the benzimidazole ring and quinoline, the alkyl radical (ethyl, cyclopropyl) at the quinoline nitrogen, and the radical at the benzimidazole nitrogen. The most potent hybrid, **141**, displayed membrane activity and did not induce bacterial resistance. Compound **141** inhibited biofilm formation and disrupted pre-established *Staphylococcus aureus* and *Escherichia coli* biofilms. Additionally, **141** inhibited the relaxation activity of *E. coli* topoisomerase IV at a concentration of 10 µM and exhibited low cytotoxicity toward mammalian cells. Molecular modeling and experimental studies suggested that **141** could effectively bind DNA, forming a stable **141**–DNA complex that may inhibit DNA replication, contributing to its potent bioactivity (Figure 12). The compound structures highlight the importance of the chlorine substituents on the phenyl ring linked to benzimidazole [85]. Arab et al. reported compound **145** as the most active antimicrobial agent among the synthesized benzimidazole–quinolones, showing an MIC of 0.097 µg/mL against *E. coli*, *S. aureus*, *S. epidermidis*, and *B. subtilis*, and outperforming Norfloxacin by 2–4 fold [86].

The structure–activity relationship (SAR) of benzimidazole–quinolinolone hybrids with antimicrobial properties can be summarized as follows:

- The methylene–piperazine bridge between the benzimidazole ring and the quinoline moiety plays a crucial role in antimicrobial activity.
- Alkyl substituents such as ethyl or cyclopropyl at the quinoline nitrogen are favorable for antimicrobial activity, whereas longer alkyl chains containing three or more carbon atoms are less effective [87].
- The presence of a methylene linker between the “2” position of the benzimidazole ring and the quinoline nitrogen is important for biological activity.
- The lipophilicity of the compounds is a key determinant of antimicrobial efficacy [88,89].
- Compounds bearing a dichlorophenyl ring exhibit higher activity than those containing only a single halogen substituent [90].
- Among the two series of compounds presented here (**98**–**103** and **109**–**144**), compounds **99** and **141** showed the best performance combined with reduced cytotoxicity. Docking studies were therefore conducted for these compounds, revealing their mechanisms of action through interactions with key residues of topoisomerase IV.

## 4. Benzimidazole-Quinoline Hybrids with Antimalarial Properties

**Scheme 9:**
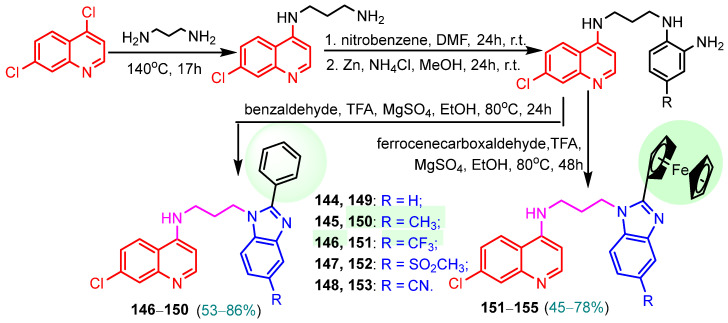
Synthesis of antimalarial hybrids **146**–**155**.

**Figure 13:**
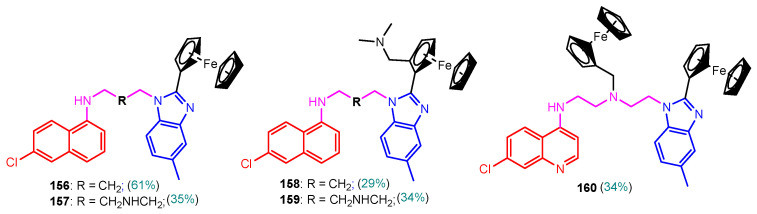
Antimalarial benzimidazole-quinolines **156**–**160**.

Malaria is a parasitic disease transmitted by mosquitoes that infect humans, posing a major challenge for global health research due to its high mortality rate. Several *Plasmodium* species are responsible for this life-threatening illness, with *Plasmodium falciparum* being the most virulent [91]. Notably, the reported antimalarial benzimidazole–quinoline hybrids incorporate ferrocenyl residues, which are of particular interest in medicinal chemistry and pharmaceutical development [92,93]. Baartzes et al. (2019) [94] synthesized two series of phenyl- and ferrocenyl-substituted aminoquinoline–benzimidazole hybrids **146**–**155** from 4,7-dichloroquinoline in four steps, as depicted in Scheme 9. All hybrids were evaluated for in vitro antiplasmodial activity against the chloroquine-sensitive NF54 and multi-drug-resistant K1 strains of *Plasmodium falciparum*. The hybrids exhibited strong antiplasmodial activity, with most IC_50_ values in the submicromolar range (Table 9). The incorporation of the ferrocenyl group enhanced the antimalarial activity of compounds **151**–**155** compared with the phenyl-substituted derivatives **146**–**150**. Compounds **148** and **150** exhibited greater potency than chloroquine, with IC_50_ values of 0.151 µM and 0.179 µM, respectively. The most active compound against the chloroquine-sensitive strain was the 2-ferrocenyl hybrid **152**, whereas the 2-phenyl hybrid **148** showed the highest activity in the resistant strain. The most active aminoquinoline–benzimidazole hybrids, **148** and **152**, were further evaluated for in vivo antimalarial efficacy in *Plasmodium berghei*-infected mice, revealing that compound **148** possesses immunomodulatory properties. Additionally, tuberculostatic activity was assessed against *Mycobacterium tuberculosis* H37Rv for the highly lipophilic phenyl-substituted hybrids **146**–**150 [94]**. Using a similar approach, Golding et al. synthesized hybrids **156**–**160** (Figure 13). These quinoline–benzimidazole ferrocenyl hybrids were evaluated for in vitro antiplasmodial activity against both the drug-sensitive Pf NF54 and multidrug-resistant Pf K1 strains using a *Plasmodium* lactate dehydrogenase (pLDH) assay [95]. The IC_50_ values and resistance indices are presented in Table 10. Notably, hybrids **155**–**157** exhibited submicromolar activity, with IC_50_ values ranging from 0.025 to 0.038 μM. The presence of a tertiary amine and an additional ferrocenyl moiety in the linker of hybrid **158** enhanced its antimalarial activity more than sixfold compared with **154**. Incorporation of a secondary amine in the linker of **155** increased its activity over 260-fold relative to its propyl-containing congener **154**, whereas the same modification in **157** compared with **156** had minimal effect on potency. Hybrids **155** and **157**, featuring an ethylenetriamine linker, displayed equipotent activity despite the presence of the side chain in complex **154**. Among the tested compounds, hybrid **155** was the most potent, exhibiting superior activity to chloroquine and enhanced selectivity toward parasitized red blood cells [96].

SAR studies of benzimidazole–quinoline hybrids with antimalarial properties indicate the following:

- The presence of a ferrocene moiety at the “2” position of the benzimidazole ring enhances antimicrobial activity [97].
- The inclusion of a second ferrocene unit in the molecule does not contribute to further increases in antimicrobial activity [98].
- The alkyl or aminoalkyl bridge between the quinoline and benzimidazole rings is essential for maintaining good antimicrobial activity [99].

## 5. Benzimidazole-Quinoline Hybrids Containing a Five-Membered Heterocyclic Ring with Antimicrobial Properties

**Scheme 10:**
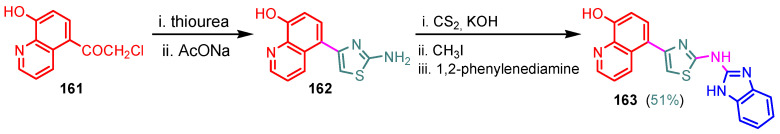
Synthesis of antimicrobial thiazole benzimidazole-quinoline hybrid **161**.

**Scheme 11:**
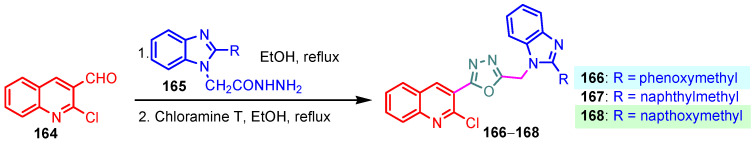
Synthesis of antimicrobial oxadiazole benzimidazole-quinoline hybrids **166**–**168**.

**Figure 14:**
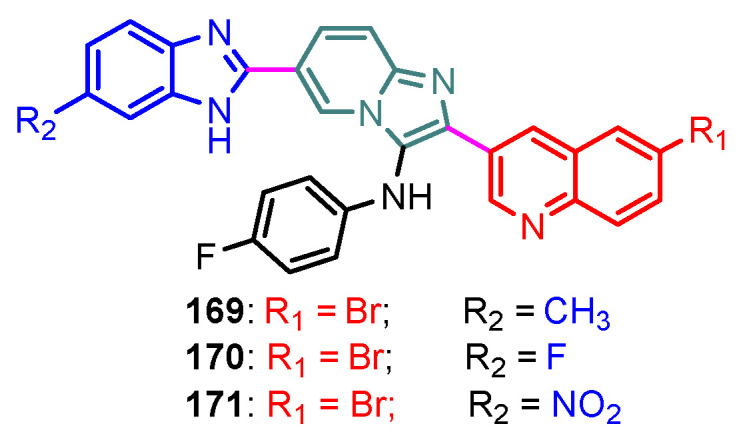
Antibacterial benzimidazole-quinoline hybrids **169**–**171**.

**Scheme 12:**
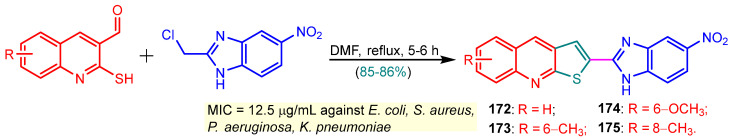
Synthesis of antimicrobial thiazole benzimidazole-quinoline hybrid **172**–**175**.

The presence of five-membered heterocycles significantly enhances the antimicrobial activity of benzimidazole–quinoline derivatives [100]. Abdel-Mohsen (2003) [101] reported the synthesis of a thiazole-linked benzimidazole–quinoline hybrid **163** from 2-chloro-1-(8-hydroxyquinolin-5-yl)ethanone **161** via the intermediate 5-(2-aminothiazol-4-yl)quinolin-8-ol **162** (Scheme 10). Hybrid **161** exhibited the strongest activity against *Bacillus subtilis* [101]. Salahuddin et al. synthesized oxadiazole-linked benzimidazole– quinoline hybrids **166**–**168** from 2-chloroquinoline-3-carbaldehyde **164** and 2-substituted 1*H*-benzo[*d*]imidazol-1-yl acetohydrazides **165** (Scheme 11). The antibacterial activities of hybrids **164**–**166** were evaluated against twelve bacterial strains, including *Shigella sonnei* E08869, *Escherichia coli* 35B, *Vibrio cholerae* 765, *Proteus vulgaris* AP169, *Bacillus subtilis* MTCC441, *Klebsiella pneumoniae* NCTC7447, *Acetobacter aceti* AP586, *Pseudomonas putida* MTCC2252, *Shigella dysenteriae* 9, *Morganella morganii* ATCC25830, *Escherichia coli* Rho7/12, and *Bacillus cereus* MTCC1305 (Table 11). Hybrid **166** demonstrated the highest antibacterial activity, exhibiting efficacy against all tested strains, with MIC values ranging from 25 to 200 µg/mL and an MIC of 25 µg/mL against *E. coli* 35B, *B. subtilis*, and *B. cereus*. In contrast, hybrid **164** was active against only half of the tested strains, with MIC values of 25 µg/mL against *B. subtilis* and *B. cereus* [102]. The incorporation of an oxadiazole ring into benzimidazole–quinoline hybrids enhanced antibacterial activity, in agreement with previous reports [103]. Al-Tel et al. (2011) synthesized imidazo[1,2-*a*]pyridine hybrids **169**–**171** via the Groebke–Blackburn reaction (Figure 14) [104]. All hybrids exhibited excellent antibacterial activity, surpassing at least one reference drug, Amoxicillin or Cefixime, and in some cases both (Table 12). Notably, compound **168** showed superior antifungal activity, with MIC values of 1.11–1.36 µg/mL compared with 2.11–9.22 µg/mL for Fluconazole [104]. Gowda et al. (2011) reported the synthesis of benzimidazole–thieno[2,3-b]quinoline hybrids **172**–**175** by refluxing a mixture of 2-(chloromethyl)-5-nitro-1*H*-benzo[*d*]imidazole and 2-mercaptoquinoline-3-carbald ehyde for 5–6 h, with yields of 85–86% (Scheme 12) [105].

The antibacterial activities of hybrids **170**–**173** against *Escherichia coli*, *Staphylococcus aureus*, *Pseudomonas aeruginosa*, and *Klebsiella pneumoniae* were 12.5 µg/mL, compared with 6.25 µg/mL for the standard Nitrofurazone [105].

SAR studies of benzimidazole–quinoline hybrids containing a five-membered heterocyclic ring with antimicrobial properties revealed the following:

- The incorporation of an oxadiazole ring into the benzimidazole–quinoline hybrids significantly enhances antibacterial activity [106].
- The presence of a phenoxymethyl group at the “2” position of benzimidazole improves antimicrobial activity more than the naphthylmethyl or naphthoxymethyl groups at the same position.
- A fluorine atom at the “6” position of benzimidazole greatly enhances its antimicrobial activity [107].

## 6. Benzimidazole-Quinoline Hybrids with Antitrypanosomal Properties

**Scheme 13:**
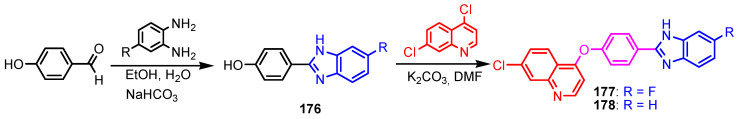
Synthesis of antitrypanosomal benzimidazole-quinoline hybrids **177** and **178**.

Infection with protozoan parasites of the genus *Trypanosoma* causes trypanosomiasis, one of several neglected tropical diseases transmitted by vectors that affect both humans and animals in tropical and subtropical regions [108,109,110,111]. Quinolones bearing fluorine substituents have been investigated for activity against trypanosomes [112]. Pomel et al. (2015) reported the synthesis of benzimidazole–quinoline hybrids **177** and **178** from 4-hydroxybenzaldehyde and 4-substituted benzene-1,2-diamines via the benzimidazole intermediate **176** (Scheme 13) [113]. Both compounds exhibited antitrypanosomal activity against *Trypanosoma brucei gambiense*, with IC_50_ values of 1.98 and 3.79 µM, respectively, which were lower than that of the standard pentamidine. Notably, compound **177** also demonstrated in vivo antitrypanosomal activity in intraperitoneally infected Swiss mice [113]. SAR studies of benzimidazole–quinoline hybrids with antitrypanosomal properties reveal that a fluorine atom at the “6” position of the benzimidazole ring significantly enhances its antimicrobial activity [114].

## 7. Benzimidazole-Quinoline Hybrid Complexes

**Scheme 14:**
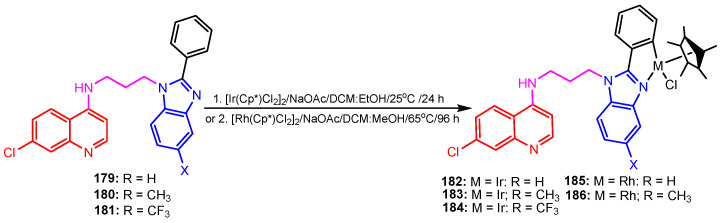
Synthesis of antimalarial benzimidazole-quinoline complexes **182**–**186**.

Heterocycle-based metal complexes, particularly those containing *N*-heterocycles, have emerged as potent antibacterial agents. These complexes often exhibit enhanced activity compared with the free ligands due to improved cellular uptake and the ability to target multiple biochemical pathways. Examples include Cu(II), Ni(II), Ru(II), Ag(I), and Au(I) complexes, many of which have demonstrated efficacy against resistant strains by disrupting essential enzymes and other vital cellular processes. Selected examples of benzimidazole–quinoline metal complexes with antimicrobial properties are presented below [115]. Baartzes et al. (2020) reported the synthesis of neutral aminoquinoline–benzimidazole complexes of Ir(III) and Rh(III) **180**–**184**, starting from the corresponding 7-chloro-N-(3-(2-phenyl-1*H*-benzo[*d*]imidazol-1-yl)propyl)quinolin-4-amine **179**–**181** and the organometallic precursors pentamethylcyclopenta dienyl iridium(III) dichloride dimer or pentamethylcyclopentadienylrhodium(III) dichloride dimer (Cp* = pentamethylcyclopentadienyl), as illustrated in Scheme 14 [116]. Table 13 shows that complexes **182**–**186** exhibit strong antiplasmodial activity in the low micromolar range.

**Scheme 15:**
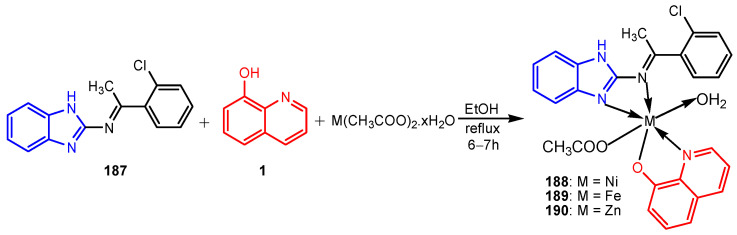
Synthesis of antimicrobial benzimidazole-quinoline complexes **188**–**190**.

The unsubstituted and methyl-substituted Ir(III) and Rh(III) complexes (**182**, **183**, **184**, and **186**) displayed similar activity against the resistant K1 strain, with IC_50_ values ranging from 1.810 to 2.844 µM. Most compounds showed comparable or slightly reduced activity in the resistant NF54 strain. Compounds **182**–**184** and **185**–**186** exhibited resistance indices (RI) between 1 and 2 (RI ≥ 1), considerably lower than that of Chloroquine (RI = 10) [116]. Seema et al. (2023) reported the synthesis of metal complexes 186–188 by refluxing a mixture of benzimidazole Schiff base **187**, quinolin-8-ol **1**, and the corresponding hydrated metal acetate in ethanol, yielding 57–72% (Scheme 15) [117]. Compared with the standard ketoconazole, Ni(II) complex **188** demonstrated excellent antifungal activity against *Aspergillus niger*, whereas complexes **189** and **190** exhibited good to moderate antibacterial activity against Gram-positive *Staphylococcus aureus* and Gram-negative *Escherichia coli* relative to the standard Ciprofloxacin [117].

SAR studies of benzimidazole–quinoline hybrid complexes indicate that complexation with iridium and nickel salts enhances their antimicrobial activity [118].

## 8. Benzimidazole-Tetrazolo[1,5-a]quinoline Hybrids with Antimicrobial Properties

**Scheme 16:**
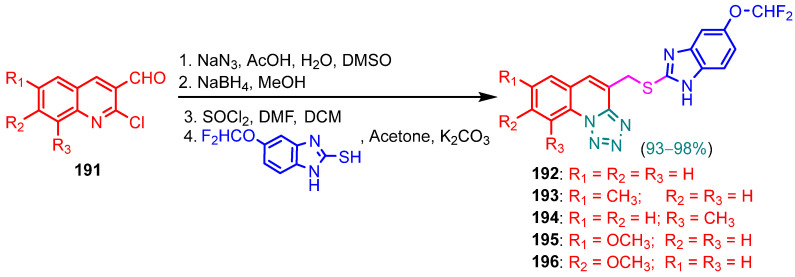
Synthesis of antimicrobial benzimidazole-tetrazolo[1,5]quinolines **192**–**196**.

**Figure 15:**
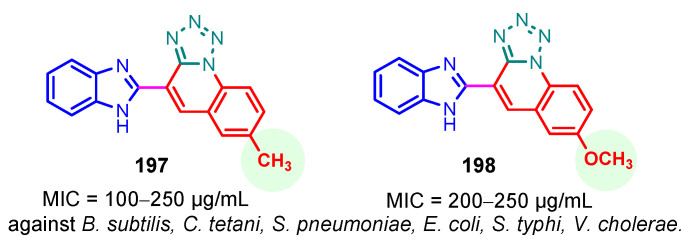
Antibacterial benzimidazole-tetrazolo[1,5]quinolines **197** and **198**.

**Scheme 17:**
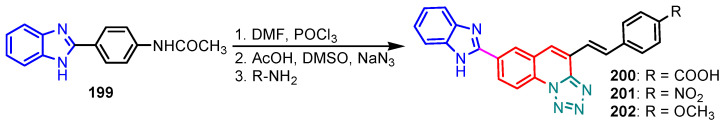
Synthesis of antimicrobial benzimidazole-tetrazolo[1,5]quinolines **198**–**200**.

Several studies have reported the synthesis of benzimidazole–tetrazolo[1,5-a]quinoline hybrids and their antimicrobial properties. Sonar et al. (2010) synthesized hybrids **190**–**194** in a four-steps process starting from quinoline **189** (Scheme 16) [119]. Compounds **190**–**194** were evaluated for antibacterial activity against Gram-positive *Bacillus subtilis* and *Staphylococcus aureus* ATCC 6538, as well as Gram-negative *Escherichia coli* ATCC 8739 and *Salmonella aboney* NCTC 6017, using Streptomycin as the reference. Although a few compounds exhibited activity against Gram-negative bacteria, most showed modest activity against Gram-positive strains (Table 14). Among the tested compounds, **194** showed the highest antibacterial activity, followed by **192** and **191** [119]. Using a similar synthetic approach, Mungra et al. (2011) prepared a series of hybrid compounds [120]. Among these, compounds **195** (methyl-substituted) and **196** (methoxy-substituted) exhibited the most potent antibacterial activity, with MIC values of 100–250 µg/mL for **197** and 200–250 µg/mL for **198** against all tested bacterial strains, including *Bacillus subtilis* MTCC441, *Clostridium tetani* MTCC449, *Streptococcus pneumoniae* MTCC1936, *Escherichia coli* MTCC443, *Salmonella typhi* MTCC98, and *Vibrio cholerae* MTCC3906 (Figure 15). The enhanced activity was attributed to methyl and methoxy substituents at the “7” position of the quinoline ring [120]. Uttarwar et al. (2013) synthesized hybrids **200**–**202** in three steps from N-(4-(1*H*-benzo[*d*]imidazol-2-yl)phenyl)acetamide **199** (Scheme 17) [121]. All compounds displayed significant antibacterial activity against *S. aureus*, *K. pneumoniae*, *B. subtilis*, and *P. aeruginosa*, as well as notable antifungal activity against *Candida albicans*, using Ciprofloxacin and Fluconazole as reference drugs [121].

The enhanced antimicrobial activity was associated with COOH, NO_2_, and OCH_3_ substituents at the “4” position of the phenyl ring [122,123,124].

SAR studies of benzimidazole–tetrazolo[1,5-a]quinoline hybrids with antimicrobial properties revealed the following:

- The presence of a methyl group at the “8” position of quinoline enhances antimicrobial activity [125], compared to the presence of methyl or methoxy substituents at the 6-position of quinoline [126].
- The inclusion of methoxy, nitro, and carboxyl groups on a phenyl ring improves antimicrobial activity [127,128].

## 9. Challenges, Limitation and Future Directions

The main challenges in synthesizing benzimidazole–quinoline hybrids are as follows:

1. Identifying new reactions that lead to benzimidazole–quinoline hybrids with reduced optimization times, while achieving good selectivity and high yields. This review highlights that many synthetic routes result in moderate yields (50–60% or lower). Several ‘one-pot’ reactions have been proposed in the literature as potential new avenues for synthesizing these hybrids [129,130]. Further studies are required to establish a correlation between the electronic effects in the molecules, the reaction mechanisms, and the yields of the desired hybrids.
2. Addressing current limitations in understanding the mode of action of benzimidazole–quinoline hybrids will require the integration of advanced computational tools capable of predicting optimal molecular structures and conformations, thereby guiding the rational design of compounds with superior antimicrobial activity.
3. As highlighted in this article, much of the existing research remains at an early, exploratory stage. Many studies report only preliminary qualitative assessments of antimicrobial activity, without comprehensive evaluation of minimum inhibitory concentrations (MICs), ADME properties, DFT calculations, in silico modeling, or in vivo validation. Consequently, substantial additional experimental and computational efforts are required to advance these initial findings toward fully developed and biologically validated antimicrobial candidates.
4. Future progress in the development of antimicrobial hybrids will depend on the consolidation of structure–activity relationship data from multiple studies into accessible and standardized databases, enabling the rational design of compounds with improved antimicrobial efficacy.
5. Achieving high solubility in antimicrobial hybrids is essential for their effectiveness and represents a key direction for future research aimed at improving the therapeutic potential of these compounds.
6. Further work is needed to explore formulations that improve their bioavailability, efficacy, and safety for potential therapeutic use.

## 10. Conclusions

This review highlights the importance of benzimidazole–quinoline hybrids, summarizing their synthetic strategies, medicinal properties, and structure–activity relationships (SAR). These compounds exhibit broad-spectrum antimicrobial activity against Gram-positive and Gram-negative bacteria and fungi, as well as notable antimalarial and antitrypanosomal effects.

The presence of specific substituents on the benzimidazole and quinoline cores—including alkyl groups (methyl, ethyl, *n*-propyl, cyclopropyl, *n*-butyl, benzyl, phenoxymethyl), halogens (-fluoro, -chloro, -bromo), hydroxyl and methoxy groups (-OH, -OCH_3_, -OCHF_2_), carbonyl and carboxyl derivatives (-CHO, -COOH, -COOCH_2_CH_3_, -CH_2_CONHNH_2_), nitro (-NO_2_), cyano (-CN), chloromethyl (-CH_2_Cl), sulfonyl (-SO_2_), as well as additional heterocycles (pyrrole, thiophene, morpholine, thiazoles, tetrazoles, oxadiazoles) and ferrocene—has been shown to enhance the antimicrobial activity of benzimidazole–quinoline hybrids. Based on the literature, several structure–activity relationships (SAR) can be identified that influence the antimicrobial activity of benzimidazole–quinoline hybrids:

- Substitution at the C “5” or C “6” positions of the benzimidazole nucleus enhances the antimicrobial activity of benzimidazole–quinoline hybrids (compounds **3**–**7**, **12**–**14**, **58**–**64**, **67**–**71**, **93**, **149**–**153**, **167**–**169**, **171**–**174**, **178**, **179**).
- Substituents at the C “2” position of the benzimidazole ring, such as quinoline (hybrid **5**), phenyl (hybrid **164**), naphthyl (hybrid **165**), or ferrocene (hybrids **154**–**158**), further increase antimicrobial activity.
- Incorporation of a methylene spacer between the benzimidazole and quinoline rings leads to enhanced antimicrobial activity (compounds **12**–**14**, **23**–**25**, **98**, **102**).
- The presence of a hydroxyl group at the C-8 position of the quinoline nucleus improves antimicrobial activity (compounds **12**, **14**, **29**).
- Chlorine substitution at the C “2” or C “5” positions of the quinoline ring is associated with increased antimicrobial activity (compounds **33**, **49**–**51**).
- Insertion of an additional heterocyclic ring between the benzimidazole and quinoline nuclei, such as thiazole (**161**), morpholine (**109**–**117**), pyrrole (**95**), thiophene (**170**–**173**), or oxadiazole (**166**), enhances the antimicrobial activity of the hybrids.
- The presence of heteroatoms in the linker connecting the two aromatic rings, including sulfur (**194**), oxygen (**33**, **35**, **37**, **50**), or an amide group (NH–CO, compound **105**), further contributes to increased antimicrobial activity.

Compound **96** demonstrated remarkable antifungal activity against 26 clinical fungal strains, surpassing the efficacy of the standard drug Fluconazole. Importantly, hybrid **96** effectively inhibited fungal growth while showing no apparent toxicity toward mammalian cells.

In silico molecular docking studies on compounds **23**, **24**, **25**, **32**, **34**, **35**, **37**, and **140** revealed favorable binding energies and multiple hydrogen bonds with key amino acid residues within the protein active-site pocket.

Currently, the literature lacks reports on ADME evaluations, nanoparticle-based formulations, or nanosystems aimed at enhancing the bioavailability of benzimidazole–quinoline hybrids, highlighting a promising direction for future research.

Overall, this review supports the rational design and synthesis of novel benzimidazole–quinoline hybrids with antimicrobial properties, particularly in the context of increasing microbial infections and the growing resistance to existing drugs.

## Figures and Tables

**Table 1:** FDA-approved benzimidazole-based drugs.

No	Drug Class	Exemple Drugs
1.	Proton Pump Inhibitors (PPIs)	Omeprazole (Prilosec)
		Pantoprazole (Protonix)
		Rabeprazole (AcipHex)
		Lansoprazole (Prevacid)
		Dexlansoprazole
		Dexrabeprazole
2.	Anthelmintics (Anti-parasitic)	Albendazole (Albenza)
		Mebendazole (Vermox)
		Thiabendazole (Mintezol)
		Triclabendazole
		Flubendazole (veterinary use)
		Fenbendazole (veterinary use)
3.	Anticancer agents	Bendamustine (Treanda)
		Binimetinib (Mektovi)
		Selumetinib (Koselugo)
4.	Antipsychotic agent	Pimozide (Orap)
5.	Anticoagulant agent	Dabigatran etexilate (Pradaxa)

**Table 2:** FDA-approved quinoline-based drugs.

No	Drug Class	Exemple Drugs
1.	Antimalarial agent	Tafenoquine (Krintafel)
2.	Antimicrobial agents	Ciprofloxacin
		Levofloxacin (Levaquin)
		Moxifloxacin (Avelox)
		Ofloxacin (Floxin)
		Norfloxacin (Noroxin)
3.	Antimycobacterial agent	Bedaquiline (Sirturo)
4.	Antiviral agent	Simeprevir (Olysio)
5.	Anticancer agents	Bosutinib (Bosulif)
		Lenvatinib (Lenvima)
		Cabozantinib (Cabometyx, Cometriq)
		Neratinib (Nerlynx)
		Capmatinib (Tabrecta)
		Tivozanib (Fotivda)
6.	Allosteric enzyme activator	Mitapivat (Pyrukynd, Aqvesme)
7.	HMG-CoA reductase inhibitor (cholesterol)	Pitavastatin (Livalo, Zypitamag)

**Table 3:** Antimicrobial activity of compounds **58**–**64**, **67**–**71** and standard drugs (zone inhibition in mm at concentration of 12.5 µg/mL).

Hybrid	Antibacterial Activity				Antifungal Activity			
	*S. aureus*	*E. coli*	*Xanthomonas* sp.	*Salmonella* sp.	*A. niger*	*A. flavus*	*Penicillium* sp.	*A. terreus*
**58**	13	13	10	9	12	1	18	14
**59**	16	12	10	13	10	17	17	9
**60**	12	17	16	28	12	13	19	10
**61**	15	15	14	15	12	10	12	9
**62**	12	11	12	11	10	10	17	14
**63**	15	12	16	12	13	15	12	9
**64**	12	16	14	13	17	1	13	9
**67**	13	16	12	15	12	12	17	14
**68**	13	16	15	17	18	16	12	16
**69**	16	16	22	12	12	10	12	11
**70**	16	11	13	11	14	14	15	12
**71**	12	14	14	11	12	10	18	13
Ciprofloxacin	20	21	22	31	–	–	–	–
Fluconazole	–	–	–	–	18	20	21	18

**Table 4:** Antimicrobial activities of compound **78** and **79**.

Compound	MIC (µg/mL)				
	*E. coli*	*B. cereus*	*S. aureus*	*C. albicans*	*A. fumigatus*
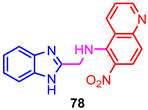	625	156.25	1250	1250	1250
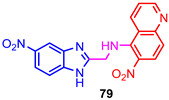	1250	312.5	2500	2500	2500
Ampicillin	19.53	1250	312.5	–	–
Fluconazole	–	–	–	2500	–

**Table 5:** Antimicrobial activity of benzimidazole-quinoline hybrids **82**–**92**.

Compound	Antibacterial Activity				Antifungal Activity	
	*S. aureus*	*B. subtilis*	*E. coli*	*P. aeruginosa*	*A. niger*	*C. albicans*
**82**	15.625	15.625	7.8	7.8	125	250
**83**	7.8	15.625	15.625	3.9	125	250
**84**	15.625	7.8	15.625	3.9	125	250
**85**	125	62.5	62.5	62.5	20.1	21.2
**86**	31.25	31.25	15.625	3.9	22.3	19.2
**87**	15.625	15.625	3.9	3.9	62.5	62.5
**88**	15.625	15.625	15.625	3.9	62.5	31.25
**89**	31.25	31.25	15.625	15.625	250	250
**90**	31.25	31.25	31.25	15.625	500	500
**91**	15.625	15.625	7.8	3.9	500	500
**92**	15.625	31.25	7.8	7.8	250	500
Ciprofloxacin	1.95	1.95	1.95	1.95	–	–
Voriconazole	–	–	–	–	15.625	15.625

Green zone—best values; yellow zone—good values; gray zone—acceptable values.

**Table 6:** MICs values of hybrid **97** and fluconazole against clinical fungal pathogens.

No	Strain	97 (µg/mL)	Fluconazole (µg/mL)
1	*C. albicans* 29434	0.25	0.5
2	*C. albicans* 29449	0.0625	0.5
3	*C. albicans* 29435	0.5	0.25
4	*C. albicans* 29448	0.25	>64
5	*C. albicans* 29437	0.0625	2
6	*C. albicans* 29446	0.5	>64
7	*C. albicans* 29453	0.0625	2
8	*C. albicans* 29438	0.25	0.5
9	*C. albicans* 29366	0.5	>64
10	*C. albicans* 29367	0.5	>64
11	*C. albicans* 29439	0.5	>64
12	*C. albicans* 29440	0.5	2
13	*C. albicans* 29441	0.5	4
14	*C. albicans* 29442	0.5	16
15	*C. albicans* 29444	0.25	2
16	*C. parapsilosis* ATCC 22019	1	0.25
17	*C. glabrata* ATCC 90030	1	1
18	*C. albicans* ATCC 10231	0.5	1
19	*C. neoformans* 41291	1	1
20	*C. neoformans* 41292	0.5	1
21	*C. neoformans* 41296	1	1
22	*C. neoformans* 41295	2	2
23	*C. neoformans* 41294	1	2
24	*C. neoformans* 41297	0.5	0.5
25	*C. neoformans* 41298	2	4
26	*C. neoformans* 41299	2	2

**Table 7:** Antibacterial activity (MIC (µg/mL)) of compounds **98**–**103**.

Compound	Gram-Positive Bacteria					Gram-Negative Bacteria					
	MRSA	*E. faecalis*	*S. aureus*	*S. aureus* ATCC25923	*S. aureus* ATCC29213	*K. pneumoniae*	*E. coli*	*P. aeruginosa*	*A. baumanii*	*P. aeruginosa* ATCC27853	*E. coli* ATCC25922
**98**	64	32	4	64	64	4	256	4	256	512	512
**99**	8	64	8	64	32	16	128	1	128	32	64
**100**	128	16	64	128	128	4	256	16	128	32	64
**101**	64	256	128	128	256	64	256	8	128	256	128
**102**	256	256	256	128	256	25	128	32	128	>512	512
**103**	8	512	4	12	128	8	512	4	512	512	512
Clinafloxacin	>512	2	0.5	0.5	0.5	32	64	32	8	32	0.5
Norfloxacin	>512	256	64	32	8	>512	>512	4	32	512	1

**Table 8:** Antibacterial activity (MIC (µg/mL)) of compounds **109**–**144** and standards.

Hybrid	Gram-Positive Bacteria				Gram-Negative Bacteria					
	MRSA	*S. aureus*	*B. subtilis*	*M. luteus*	*E. coli* DH52	*E. coli* JM109	*S. dysenteriae*	*P. aeruginosa*	*B. proteus*	*S. enterica*
**109**	4 ± 0.58	2 ± 0.23	2 ± 0.23	4 ± 0.58	2 ± 0.23	8 ± 1.15	4 ± 0.58	2 ± 0.23	1 ± 0.12	2 ± 0.23
**110**	0.5 ± 0.05	1 ± 0.12	2 ± 0.23	1 ± 0.12	1 ± 0.12	2 ± 0.23	0.5 ± 0.05	0.5 ± 0.05	2 ± 0.23	1 ± 0.12
**111**	1 ± 0.12	2 ± 0.23	1 ± 0.12	1 ± 0.12	0.5 ± 0.05	4 ± 0.58	2 ± 0.23	1 ± 0.12	0.5 ± 0.05	0.5 ± 0.05
**112**	16 ± 1.73	16 ± 1.73	8 ± 1.15	0.5 ± 0.05	16 ± 1.73	32 ± 3.46	8 ± 1.15	4 ± 0.58	2 ± 0.23	8 ± 1.15
**113**	1 ± 0.12	2 ± 0.23	16 ± 1.73	1 ± 0.12	0.5 ± 0.05	2 ± 0.23	1 ± 0.12	1 ± 0.12	0.5 ± 0.05	0.5 ± 0.05
**114**	32 ± 3.46	64 ± 6.35	8 ± 1.15	2 ± 0.23	16 ± 1.73	16 ± 1.73	16 ± 1.73	2 ± 0.23	4 ± 0.58	8 ± 1.15
**115**	8 ± 1.15	16 ± 1.73	8 ± 1.15	1 ± 0.12	8 ± 1.15	32 ± 3.46	4 ± 0.58	2 ± 0.23	2 ± 0.23	4 ± 0.58
**116**	0.125 ± 0.01	0.25 ± 0.02	0.125 ± 0.01	0.125 ± 0.01	0.125 ± 0.01	0.5 ± 0.05	1 ± 0.12	0.5 ± 0.05	0.125 ± 0.01	0.125 ± 0.01
**117**	2 ± 0.23	4 ± 0.58	0.5 ± 0.05	2 ± 0.23	1 ± 0.12	4 ± 0.58	2 ± 0.23	2 ± 0.23	1 ± 0.12	1 ± 0.12
**118**	0.5 ± 0.05	0.5 ± 0.05	0.5 ± 0.05	0.25 ± 0.02	0.5 ± 0.05	0.5 ± 0.05	0.5 ± 0.05	0.5 ± 0.05	0.5 ± 0.05	0.5 ± 0.05
**119**	4 ± 0.58	64 ± 6.35	16 ± 1.73	16 ± 1.73	8 ± 1.15	32 ± 3.46	16 ± 1.73	1 ± 0.12	8 ± 1.15	4 ± 0.58
**120**	2 ± 0.23	16 ± 1.73	8 ± 1.15	8 ± 1.15	4 ± 0.58	8 ± 1.15	4 ± 0.58	0.5 ± 0.05	4 ± 0.58	4 ± 0.58
**121**	8 ± 1.15	128 ± 68.65	2 ± 0.23	4 ± 0.58	4 ± 0.58	32 ± 3.46	128 ± 68.65	32 ± 3.46	1 ± 0.12	0.5 ± 0.05
**122**	16 ± 1.73	256 ± 137.29	2 ± 0.23	8 ± 1.15	32 ± 3.46	128 ± 68.65	32 ± 3.46	32 ± 3.46	128 ± 68.65	16 ± 1.73
**123**	64 ± 6.35	512 ± 49.65	32 ± 3.46	16 ± 1.73	8 ± 1.15	128 ± 68.65	128 ± 68.65	64 ± 6.35	8 ± 1.15	256 ± 137.29
**124**	32 ± 3.46	256 ± 137.29	16 ± 1.73	4 ± 0.58	4 ± 0.58	64 ± 6.35	32 ± 3.46	16 ± 1.73	4 ± 0.58	8 ± 1.15
**125**	2 ± 0.23	32 ± 3.46	32 ± 3.46	4 ± 0.58	8 ± 1.15	32 ± 3.46	16 ± 1.73	2 ± 0.23	4 ± 0.58	4 ± 0.58
**126**	8 ± 1.15	32 ± 3.46	4 ± 0.58	16 ± 1.73	0.5 ± 0.05	32 ± 3.46	8 ± 1.15	4 ± 0.58	8 ± 1.15	8 ± 1.15
**127**	128 ± 68.65	256 ± 137.29	0.5 ± 0.05	2 ± 0.23	2 ± 0.23	128 ± 68.65	8 ± 1.15	2 ± 0.23	0.5 ± 0.05	0.5 ± 0.05
**128**	2 ± 0.23	32 ± 3.46	32 ± 3.46	1 ± 0.12	1 ± 0.12	4 ± 0.58	4 ± 0.58	1 ± 0.12	32 ± 3.46	4 ± 0.58
**129**	64 ± 6.35	128 ± 68.65	4 ± 0.58	4 ± 0.58	1 ± 0.12	128 ± 68.65	8 ± 1.15	2 ± 0.23	2 ± 0.23	4 ± 0.58
**130**	128 ± 68.65	256 ± 137.29	16 ± 1.73	32 ± 3.46	16 ± 1.73	64 ± 6.35	64 ± 6.35	16 ± 1.73	16 ± 1.73	16 ± 1.73
**131**	1 ± 0.12	16 ± 1.73	32 ± 3.46	1 ± 0.12	0.5 ± 0.05	2 ± 0.23	4 ± 0.58	1 ± 0.12	0.5 ± 0.05	4 ± 0.58
**132**	64 ± 6.35	64 ± 6.35	64 ± 6.35	128 ± 68.65	128 ± 68.65	128 ± 68.65	64 ± 6.35	32 ± 3.46	32 ± 3.46	32 ± 3.46
**133**	64 ± 6.35	32 ± 3.46	4 ± 0.58	4 ± 0.58	8 ± 1.15	16 ± 1.73	2 ± 0.23	4 ± 0.58	4 ± 0.58	16 ± 1.73
**134**	64 ± 6.35	64 ± 6.35	64 ± 6.35	128 ± 68.65	128 ± 68.65	128 ± 68.65	64 ± 6.35	32 ± 3.46	64 ± 6.35	16 ± 1.73
**135**	0.125 ± 0.01	8 ± 1.15	16 ± 1.73	0.5 ± 0.05	2 ± 0.23	16 ± 1.73	16 ± 1.73	1 ± 0.12	4 ± 0.58	0.5 ± 0.05
**136**	2 ± 0.23	64 ± 6.35	16 ± 1.73	2 ± 0.23	8 ± 1.15	64 ± 6.35	64 ± 6.35	2 ± 0.23	32 ± 3.46	4 ± 0.58
**137**	16 ± 1.73	8 ± 1.15	8 ± 1.15	8 ± 1.15	32 ± 3.46	32 ± 3.46	16 ± 1.73	8 ± 1.15	32 ± 3.46	8 ± 1.15
**138**	0.125 ± 0.01	4 ± 0.58	8 ± 1.15	0.125 ± 0.01	1 ± 0.12	4 ± 0.58	2 ± 0.23	1 ± 0.12	1 ± 0.12	0.25 ± 0.02
**139**	8 ± 1.15	4 ± 0.58	2 ± 0.23	4 ± 0.58	16 ± 1.73	8 ± 1.15	4 ± 0.58	4 ± 0.58	16 ± 1.73	4 ± 0.58
**140**	4 ± 0.58	16 ± 1.73	0.5 ± 0.05	4 ± 0.58	2 ± 0.23	16 ± 1.73	16 ± 1.73	2 ± 0.23	1 ± 0.12	2 ± 0.23
**141**	0.125 ± 0.01	8 ± 1.15	4 ± 0.58	0.0625 ± 0.007	0.125 ± 0.01	1 ± 0.12	1 ± 0.12	0.5 ± 0.05	0.0312 ± 0.005	0.0312 ± 0.005
**142**	2 ± 0.23	4 ± 0.58	4 ± 0.58	8 ± 1.15	16 ± 1.73	8 ± 1.15	8 ± 1.15	4 ± 0.58	8 ± 1.15	16 ± 1.73
**143**	0.5 ± 0.05	2 ± 0.23	8 ± 1.15	4 ± 0.58	1 ± 0.12	16 ± 1.73	0.5 ± 0.05	1 ± 0.12	8 ± 1.15	2 ± 0.23
**144**	0.25 ± 0.02	1 ± 0.12	1 ± 0.12	1 ± 0.12	1 ± 0.12	4 ± 0.58	0.25 ± 0.02	0.5 ± 0.05	1 ± 0.12	1 ± 0.12
Chloromicin	16 ± 1.73	16 ± 1.73	32 ± 3.46	8 ± 1.15	32 ± 3.46	32 ± 3.46	16 ± 1.73	32 ± 3.46	32 ± 3.46	32 ± 3.46
Norfloxacin	8 ± 1.15	2 ± 0.23	4 ± 0.58	2 ± 0.23	1 ± 0.12	1 ± 0.12	16 ± 1.73	16 ± 1.73	8 ± 1.15	4 ± 0.58
Ciprofloxacin	2 ± 0.23	0.5 ± 0.05	2 ± 0.23	2 ± 0.23	2 ± 0.23	2 ± 0.23	2 ± 0.23	1 ± 0.12	2 ± 0.23	1 ± 0.12
Clinafloxacin	1 ± 0.12	0.5 ± 0.05	0.5 ± 0.05	0.5 ± 0.05	0.5 ± 0.05	0.5 ± 0.05	0.5 ± 0.05	0.5 ± 0.05	0.5 ± 0.05	0.25 ± 0.02

**Table 9:** In vitro antiplasmodial IC_50_ values and resistance indices of hybrids **146**–**155**.

Compound	IC_50_ (µM) ± SEM		RI ^a^
	NF54	KI	
**146**	5.55 ± 0.0627	0.201 ± 0.00268	0.0362
**147**	0.784 ± 0.0163	0.457 ± 0.0945	0.583
**148**	0.431 ± 0.0115	0.151 ± 0.00351	0.350
**149**	0.559 ± 0.0105	1.83 ± 0.0797	3.28
**150**	0.975 ± 0.0174	0.179 ± 0.00365	0.183
**151**	2.88 ± 0.0479	0.339 ± 0.0113	0.118
**152**	0.329 ± 0.005	0.283 ± 0.00376	0.860
**153**	1.22 ± 0.0996	0.658 ± 0.0392	0.538
**154**	0.848 ± 0.0069	0.579 ± 0.00770	0.683
**155**	0.909 ± 0.0194	0.565 ± 0.00751	0.622
Chloroquine	0.0102 ± 0.0023	0.205 ± 0.00709	20.20
Artesunate	0.0098 ± 0.0011	not determined	–

^a^ RI = IC_50_(K1)/IC_50_(NF54).

**Table 10:** In vitro antiplasmodial IC_50_ values and resistance indices of hybrids **156**–**160**.

Compound	IC_50_ (µM) ± SE			RI ^a^
	*Pf* NF54 IC_50_ ± SEM ^b^ (μM)	*Pf* K1 IC_50_ ± SEM (μM) ^b^	CC_50_ (CHO) ± SEM (μM) ^c^	
**156**	9.91 ± 0.71	6.29 ± 0.51	43.7 ± 2.5	0.63
**157**	0.038 ± 0.002	0.0094 ± 0.0009	>50	0.25
**158**	0.025 ± 0.001	0.113 ± 0.005	6.1 ± 1.2	4.4
**159**	0.038 ± 0.003	0.17 ± 0.02	29.8 ± 0.5	4.5
**160**	1.51 ± 0.14	14.47 ± 1.14	>50	9.6
Chloroquine	0.015 ± 0.001	0.17 ± 0.01	not determined	11.3
Ferroquine	0.0020 ± 0.003	0.0239 ± 0.0002	not determined	1.20

^a^ Results are the mean ± SEM obtained from samples screened in technical duplicate, over three or four biological replicates. ^b^ Results are the mean ± SEM obtained from samples screened in technical triplicate on a single occasion. ^c^ RI = IC_50_(K1)/IC_50_(NF54).

**Table 11:** Antibacterial activity (MIC (µg/mL)) of compounds **166**–**168** and standard.

Hybrid	*S.* *sonnei*	*E. coli* 35B	*V.* *cholerae*	*P.* *vulgaris*	*B.* *subtilis*	*K.* *pneumoniae*	*A. aceti*	*P. putida*	*S.* *dysenteriae*	*M.* *morganii*	*E. coli* Rho7/12	*B. cereus*
**166**	–	200	–	–	25	–	–	100	>200	100	100	25
**167**	>200	100	100	50	100	100	50	50	100	50	–	–
**168**	100	25	200	100	25	50	50	100	200	100	50	25
Ciprofloxacin	12.5	12.5	12.5	12.5	12.5	12.5	12.5	12.5	12.5	12.5	12.5	12.5

**Table 12:** Antibacterial activity (MIC (µg/mL)) of compounds **169**–**1671**.

Compound	Gram-Positive Bacteria			Gram-Negative Bacteria		
	*S. aureus*	*E. faecalis*	*B. megaterium*	*E. coli*	*P. aeruginosa*	*E. aerogenes*
**169**	7.43 ± 1.15	7.34 ± 1.21	7.54 ± 1.12	6.21 ± 1.12	5.45 ± 0.92	5.35 ± 0.77
**170**	1.46 ± 0.01	1.47 ± 0.04	2.53 ± 1.01	1.34 ± 0.04	1.35 ± 0.06	1.55 ± 0.02
**171**	2.12 ± 0.26	3.36 ± 0.18	4.12 ± 0.53	2.14 ± 0.11	2.32 ± 0.15	3.15 ± 0.10
Amoxicillin	12.92 ± 1.32	1.62 ± 0.03	2.28 ± 0.12	15.32 ± 1.12	14.67 ± 1.11	3.45 ± 0.21
Cefixime	34.64 ± 2.32	28.36 ± 1.32	126.32 ± 4.35	2.12 ± 0.01	16.43 ± 1.21	27.13 ± 1.33

**Table 13:** In vitro antiplasmodial IC_50_ values and resistance indices RI of ligands **179**–**181** and hybrids **182**–**186**.

Compound	IC_50_ (µM) ± SE		RI
	NF54	K1	
**179**	5.553 ± 0.63	0.201 ± 0.003	0.036
**180**	0.784 ± 0.016	0.457 ± 0.95	0.583
**181**	0.431 ± 0.012	0.151 ± 0.004	0.350
**182**	2.007 ± 0.075	2.844 ± 0.030	1.417
**183**	1.676 ± 0.171	2.181 ± 0.025	1.301
**184**	0.488 ± 0.062	0.688 ± 0.32	1.410
**185**	1.073 ± 0.028	2.217 ± 0.167	2.066
**186**	1.327 ± 0.255	1.810 ± 0.176	1.364
Chloroquine	0.016 ± 0.001	0.164 ± 0.018	10.250

**Table 14:** Antibacterial activity of hybrids **192**–**196** and standard drug (zone inhibition in mm).

Hybrid	Gram Positive				Gram Negative			
	*Bacillus subtilis*		*Staphylococcus aureus* ATCC6538		*Escherichia coli* ATCC8739		*Salmonella aboney* NCTC6017	
	Concentration (mg/mL)							
	10	20	10	20	10	20	10	20
**192**	12	15	9	11	10	12	11	14
**193**	11	13	10	14	14	15	13	16
**194**	13	16	9	13	15	17	11	13
**195**	10	13	7	10	13	15	10	13
**196**	12	16	10	15	17	19	14	17
Streptomycin	18	–	19	–	22	–	20	–

## Data Availability

No new data were created or analyzed in this study. Data sharing is not applicable to this article.

## Abbreviations

AcOH	Acetic acid
AcONa	Sodium acetate
DCM	Dichloromethan
DMF	Dimethylformamid
DMSO	Dimethylsulfoxid
Et	Ethyl
EtOH	Ethanol
Me	Methyl
MeOH	Methanol
MIC	Minimum inhibitory concentration
PPA	Polyphosphoric acid
Ph	Phenyl
Py	Pyridine
SAR	Structure–activity relationship
TBTU	2-(1*H*-Benzotriazol-1-yl)-1,1,3,3-tetramethyluronium tetrafluoroborate
*t-*BuOK	Potassium *tert*-butoxide
